# COVID-19 and *Clostridioides difficile* Coinfection Outcomes among Hospitalized Patients in the United States: An Insight from National Inpatient Database

**DOI:** 10.3390/idr15030028

**Published:** 2023-05-19

**Authors:** Rehmat Ullah Awan, Karthik Gangu, Anthony Nguyen, Prabal Chourasia, Oscar F. Borja Montes, Muhammad Ali Butt, Taimur Sohail Muzammil, Rao Mujtaba Afzal, Ambreen Nabeel, Rahul Shekhar, Abu Baker Sheikh

**Affiliations:** 1 Department of Internal Medicine, Ochsner Rush Medical Center, Meridian, MS 39301, USA; 2 Department of Internal Medicine, University of Kansas Medical Center, Kansas City, KS 66160, USA; 3 Division of Internal Medicine, University of New Mexico Health Sciences Center, Albuquerque, NM 87106, USA; 4 Department of Hospital Medicine, Mary Washington Hospital, Fredericksburg, VA 22401, USA; 5 Department of Internal Medicine, Allegheny Health Network, Pittsburgh, PA 15212, USA; 6 Department of Internal Medicine, University of Pittsburg Medical Center, Pittsburgh, PA 15213, USA

**Keywords:** COVID-19, complications, mortality, national inpatient sample, *Clostridioides difficile*

## Abstract

The incidence of *Clostridioides difficile* infection (CDI) has been increasing compared to pre-COVID-19 pandemic levels. The COVID-19 infection and CDI relationship can be affected by gut dysbiosis and poor antibiotic stewardship. As the COVID-19 pandemic transitions into an endemic stage, it has become increasingly important to further characterize how concurrent infection with both conditions can impact patient outcomes. We performed a retrospective cohort study utilizing the 2020 NIS Healthcare Cost Utilization Project (HCUP) database with a total of 1,659,040 patients, with 10,710 (0.6%) of those patients with concurrent CDI. We found that patients with concurrent COVID-19 and CDI had worse outcomes compared to patients without CDI including higher in-hospital mortality (23% vs. 13.4%, aOR: 1.3, 95% CI: 1.12–1.5, *p* = 0.01), rates of in-hospital complications such as ileus (2.7% vs. 0.8%, *p* < 0.001), septic shock (21.0% vs. 7.2%, aOR: 2.3, 95% CI: 2.1–2.6, *p* < 0.001), length of stay (15.1 days vs. 8 days, *p* < 0.001) and overall cost of hospitalization (USD 196,012 vs. USD 91,162, *p* < 0.001). Patients with concurrent COVID-19 and CDI had increased morbidity and mortality, and added significant preventable burden on the healthcare system. Optimizing hand hygiene and antibiotic stewardship during in-hospital admissions can help to reduce worse outcomes in this population, and more efforts should be directly made to reduce CDI in hospitalized patients with COVID-19 infection.

## 1. Introduction

The COVID-19 pandemic has placed significant burdens on healthcare systems worldwide, including economic challenges related to increased healthcare costs, and a higher incidence of in-hospital complications such as nosocomial infections. One such nosocomial infection is *Clostridioides difficile* infection (CDI), one of the most reported healthcare-associated infections. The incidence of CDI has been reported to increase since the onset of the COVID-19 pandemic [1]. Several mechanisms have been proposed to explain the association between COVID-19 and CDI, including gut dysbiosis, non-adherence to hand hygiene protocols, and poor antibiotic stewardship [2,3,4].

The increased use of antibiotics during the COVID-19 pandemic, particularly for treating bacterial co-infections and as a prophylactic measure, may have contributed to the higher incidence of CDI and the emergence of more resistant strains of *Clostridioides difficile*. Antibiotic overuse disrupts the normal gut flora, which can lead to an increased risk of CDI and facilitate the spread of antibiotic-resistant strains [2]. While much of the literature has attributed the increased incidence of CDI to the COVID-19 pandemic, some studies suggest a weaker association between the two, and proposed that the observed increase in CDI case incidence may be due to reduced testing [5]. Comparisons between the pre-COVID era and the COVID-19 pandemic have revealed a positive correlation between these two conditions; however, the significance of this trend remains uncertain [6]. Considering the current understanding of the link between antibiotic use and CDI, careful evaluation of antibiotic administration in COVID-19 patients is recommended. Antibiotics should not be prescribed outside of clinical settings unless there is strong evidence of a bacterial superinfection, such as the reappearance of fever, new-onset pneumonia evident on radiological images, or microbiological evidence of a bacterial infection [7]. A reduction in the current overuse of antibiotics could potentially control antibiotic resistance and side effects, including CDI, during the COVID-19 pandemic [7].

With data available since the start of the pandemic, it is crucial to analyze the relationship between COVID-19 and CDI to better understand their interplay and inform strategies for mitigating CDI. As a preventable condition, proper management of CDI can lead to reduced healthcare costs and improved patient outcomes. In this study, we aimed to examine the characteristics and outcomes of COVID-19 patients with and without CDI using the National Inpatient Sample (NIS) 2020 data from the United States to provide insights into the association between these two conditions and inform future prevention and treatment efforts.

## 2. Methods

This retrospective study utilized the Agency for Healthcare and Research and Quality (AHRQ) sponsored National Inpatient Sample (NIS) Healthcare Cost Utilization Project (HCUP) database, which is an all-payer database that approximates a 20% stratified sample of discharges from US community hospitals. Specifically, this analysis used the 2020 NIS dataset, which included hospitalization from 1 January 2020 to 31 December 2020, and was made available to the public in October of 2022 [8]. The NIS database contains data regarding in-hospital outcomes, procedures, and other discharge-related information. All patients 18 years of age and older and admitted to the hospital with COVID-19 infection were included in this study. Patients were then divided into two cohorts based on the presence of CDI. International classification of diseases 10th—clinical modification (ICD-10-CM) codes were used to retrieve the patient samples with comorbid conditions, and ICD-10 procedure codes were used to identify inpatient procedures. A detailed code summary is provided in Supplementary Table S1. Patients who were under the age of 18 years, or were transferred out of the index hospital, were excluded from our study.

The NIS database contains a de-identified collection of billing and diagnostic codes from participating hospitals. The NIS dataset does not involve ‘human subjects’ directly (consistent with federal regulations and guidance [9]), and is therefore exempt from institutional review board approval.

### 2.1. Covariates

The NIS database contains data regarding in-hospital outcomes, procedures, and other discharge-related information. Variables were divided into patient-related, hospital-related, and indicators of illness severity, which were as follows:

- Patient characteristics: age, race, sex, comorbidities, insurance status, mean income in patient’s zip code, and disposition.
- Hospital: location, teaching status, bed size, and region.
- Illness severity: length of stay, mortality, hospitalization cost, and Elixhauser comorbidity score.
- In-hospital complications: as detailed below.

### 2.2. Study Outcomes

The primary outcome was in-hospital mortality. Secondary outcomes were septic shock, acute kidney injury, acute kidney disease on hemodialysis, mechanical ventilation, ascites, peritonitis, intestinal perforation, ileus, electrolyte imbalance, mean total hospital charge, mean length of hospital stay, and disposition. 

### 2.3. Statistical Methods

STATA 17 (StataCorp LLC, College Station, TX, USA) was utilized for statistical analysis. Patients who were hospitalized with COVID-19 infection were retrieved from the NIS database using ICD-10-CM codes and were subsequently divided into two cohorts based on their COVID-19 status. The described patient characteristics, hospital characteristics, primary and secondary outcomes, and in-hospital complications were then extracted for each patient.

Chi-squared analysis was used to compare the categorical variables, while linear regression was performed to compare continuous variables among both study cohorts (CDI with COVID-19 vs. COVID-19 without CDI). For our primary outcome, in-hospital mortality, we utilized a multivariate logistic regression model to adjust for potential confounders; a two-tailed *p*-value of less than 0.05 was considered statistically significant. This multivariate logistic regression model was built using covariates with unadjusted *p*-values of less than 0.2 on initial univariate regression. We arbitrarily chose a *p*-value of less than 0.2 for covariate inclusion to broaden the number of variables accounted for by our regression model, and to decrease the probability of missing potential confounders that may impact the standard error for our exposure variable. Our multivariate analysis adjusted for sex, age, race, median household income, insurance status, hospital geographic division, Elixhauser comorbidity score, and numerous other in-hospital complications. Similarly, for our other secondary continuous outcomes, being the length of stay and cost of hospitalization, multivariate linear regression was employed; a two-tailed *p*-value of 0.05 was considered statistically significant. We conducted a secondary analysis with propensity score matching (PSM) to confirm the results obtained by the traditional multivariate analysis. Baseline demographics (age, race, sex, income status, and insurance status) were matched using a 1:1 nearest neighbor propensity score with 0.05 caliper width n matched cohort, and a secondary multivariate regression model was built as described above (Supplementary Figure S1).

## 3. Results

### 3.1. Baseline Characteristics

A total of 1,659,040 patients were hospitalized with COVID-19 infection between 1 January 2020 and 31 December 2020. Of these patients, 10,710 (0.6%) patients had concurrent CDI. We found that among the COVID-19 patients, CDI was more prevalent among females (52.4% vs. 47.9%, *p* = 0.001), Caucasians (59.5% vs. 50.9%, *p* < 0.001), followed by African Americans (20.7% vs. 19.0%, *p* < 0.001), patients aged 70 years and older (55.7% vs. 40.9%, *p* < 0.001), followed by 50–69 years (33.8% vs. 37.3%). COVID-19 patients with CDI did not reveal any significant differences in income levels (Table 1), although most of the patients were insured under Medicare (72.2% vs. 53.1%, *p* < 0.001) when compared to COVID-19 patients without CDI (Table 1). Although patients were widely distributed among various geographical locations, most COVID-19 patients, both with and without CDI, were admitted to urban teaching hospitals (73.5% and 71.5%). The baseline characteristics of both study cohorts are outlined in Table 1. COVID-19 patients with CDI were more likely to have pre-existing coronary artery disease (CAD) (23.3% vs. 17.9%, *p* < 0.001), congestive heart failure (CHF) (31.6% vs. 17.5%, *p* < 0.001), hypertension (HTN) (48.7% vs. 27.1%, *p* < 0.001), diabetes mellitus (DM) (46.6% vs. 39.8%, *p* < 0.001), chronic liver disease (9.0% vs. 5.4%, *p* < 0.001), cancer (7.6% vs. 3.5%, *p* < 0.001), and chronic kidney disease (CKD) (41.5% vs. 20.6%, *p* < 0.001) when compared to COVID-19 patients without CDI. Conversely, obesity was seen more in patients without CDI (19.3% vs. 25.7%, *p* < 0.001). The baseline characteristics of the matched cohort after the propensity matching are provided in Supplementary Table S2.

### 3.2. In-Hospital Mortality

After multivariant adjustment, we found that the in-hospital mortality was significantly higher among COVID-19 patients with CDI in comparison to COVID-19 patients without CDI (23% vs. 13.4%, aOR: 1.3, 95% CI: 1.2–1.5, *p* = 0.01) (Table 2). We conducted a subgroup analysis of mortality and found that among CDI patients with COVID-19, females had a higher in-hospital mortality rate compared to COVID-19 patients without CDI (50.1% vs. 41.4%, *p* < 0.01). Furthermore, African Americans (20.9% vs. 16.8%, *p* = 0.02) and Caucasians (57.8% vs. 57.1%, *p* = 0.006) were found to have higher in-hospital mortality rates compared to their counterparts in the COVID-19 without CDI cohort. Conversely, Hispanics in the COVID-19 with CDI cohort had a significantly lower proportion of in-hospital mortality compared to Hispanics in the COVID-19 without CDI cohort (13.2% vs. 19.7%, *p* = 0.002). There were no statistical differences in mortality among age groups found in both cohorts. Table 3 outlines the subgroup analysis of mortality.

After PSM, there were a total of 10,020 patients in each group (with CDI and without CDI) (Supplementary Table S2). The in-hospital mortality (*n* = 2325) was found to have remained significantly higher in CDI patients with COVID-19 compared to those without CDI (*n* = 1710) 23.2% vs. 17.1%, aOR: 1.2 [95% CI 1.02–1.65], *p* = 0.02) (Table 4).

### 3.3. In-Hospital Complications

COVID-19 patients with CDI were more likely to develop ileus (2.7% vs. 0.8%, *p* < 0.001), peritonitis (0.8% vs. 0.2%, *p* < 0.001), electrolyte disturbances (74.7% vs. 50.2%, *p* < 0.001), intestinal perforation (0.4% vs. 0.1%, *p* < 0.001), and ascites (2.5% vs. 0.5%, *p* < 0.001). Moreover, patients with CDI also developed septic shock more often (21.0% vs. 7.2%, aOR: 2.3, 95% CI: 2.1–2.6, *p* < 0.001), acute kidney injury (AKI) (47.7% vs. 28.5%, aOR: 1.5, 95% CI: 1.3–1.6, *p* < 0.001), and AKI requiring hemodialysis (HD) (7.7% vs. 2.4%, aOR: 2.0, 95% CI: 1.7–2.4, *p* < 0.001), and these patients also required more mechanical ventilation (28.0% vs. 15.8%, aOR: 1.4, 95% CI: 1.3–1.6, *p* < 0.001) as compared to those without CDI.

After PSM, patients with CDI and COVID-19 required more mechanical ventilation (27.8% vs. 16.9%, aOR: 1.4 [95% CI 1.1–1.9], *p* = 0.01). Higher rates of septic shock (21.1% vs. 9.1%, aOR: 2.3 [95% CI 1.6–3.2], *p* < 0.001), acute kidney injury (47.7% vs. 34%, aOR: 1.6 [95% CI 1.2–2], *p* < 0.001) and acute kidney injury requiring dialysis (7.5% vs. 2.4%, aOR: 2.2 [1.2–3.9], *p* < 0.001] were consistently observed in this cohort (Table 4).

### 3.4. In-Hospital Quality Measures and Disposition

COVID-19 patients with CDI had an increased length of stay (15.1 days vs. 8 days, adjusted length of stay 5.5 days higher, *p* < 0.001) and experienced a higher cost of hospitalization (USD 196,012 vs. USD 91,162, adjusted total charge USD 80,602 higher, *p* < 0.001) when compared to COVID-19 patients without CDI.

Patients with CDI were less likely to be discharged directly home (28.1% vs. 61.2%, *p* < 0.001), and most were discharged to either a skilled nursing facility (SNF), long-term acute care (LTAC), or nursing home (NH) (49.7% vs. 22%, *p* < 0.001). Of the patients that did get discharged home, a larger proportion required home healthcare services (21.2% vs. 15.4%, *p* < 0.001) (Table 2).

After PSM, CDI patients with COVID-19-positive continued to have an increased mean length of stay (15.1 days vs. 8.5 days, adjusted length of stay 6.4 days higher, *p* < 0.001) and a higher mean total hospitalization charge (USD 193,014 vs. USD 666,954, adjusted total charge USD 99,456 higher, *p* < 0.001) than COVID-19 patients without CDI (Table 4).

## 4. Discussion

Our study made the following key conclusions: (1) COVID-19 patients with CDI had a significantly increased in-hospital mortality when compared to COVID-19 patients without CDI, (2) COVID-19 patients with CDI had a higher complication rate including ileus, viscus perforation, septic shock, AKI, AKI requiring HD, and mechanical ventilation, (3) COVID-19 patients with CDI had a higher length of stay leading to increased total hospitalization charges, and (4) Caucasian and African American patients with COVID-19 with CDI had higher in-hospital mortality rates. To our knowledge, our study is the largest to analyze in-hospital trends in patients admitted with COVID-19 infection and concomitant CDI. Literature reporting prevalence of enteric infections in COVID-19 patients were found to be around 0.9% which is close to that found in our study at 0.64%, however we exclusively investigated exclusively CDI [5]. From a cellular level, it was postulated that COVID-19 viral infection can lead to impaired gut immunity and dysbiosis predisposing to CDI, whereas the inappropriate use of antibiotics during the pandemic is also a primary risk factor for CDI [3,4].

Our study is the first to report mortality amongst the COVID-19 and CDI cohort at 23%, which is close to 25.1% reported in a study analyzing patients with CDI on mechanical ventilators [10]. The high mortality reported in the CDI cohort was likely due to a higher prevalence of chronic medical conditions in this group (Table 1). Moreover, it is safe to deduce that the higher the patient acuity in COVID-19 cases, the higher the chances of contracting CDI due to host vulnerability.

Patients with COVID-19 and concomitant CDI were also observed to have a higher prevalence of essential hypertension (HTN), coronary artery disease (CAD) and congestive heart failure (CHF). A meta-analysis showed that preexisting CAD is roughly present in a tenth of patients hospitalized for COVID-19 [11]. Similarly, CHF and CAD are overall associated with an increased mortality during active COVID-19 infection [12,13]. Furthermore, a large cohort studying more than 150,000 cardiac patients identified CDI to be the most common healthcare-associated infection, accounting up to 75% of total infections [14]. Another large-scale study comprising >5 million patients had a similar conclusion, where heart failure was associated with higher rates of CDI leading to increased mortality [15]. The positive correlation of CDI between CHF and CAD could be due to intestinal vascular congestion leading to changes in the gut microbiome, and an additionally poor nutritional status in heart failure patients also predisposes them to CDI [16]. To further add to the medical literature, we analyzed COVID-19 and CDI in combination, and saw that CHF, CAD, and HTN were more common in the CDI cohort, further reinforcing our findings in accordance with available medical literature.

*C. difficile* is a Gram-positive, spore-forming, anaerobic bacterium that is ubiquitous in the environment, being carried by 7% of healthy adults [17]. As chronic hyperglycemia affects both innate and humoral immunity, diabetic patients are at a higher risk of developing complications from COVID-19 and CDI [18,19]. Diabetes is frequently observed in hospitalized COVID-19 patients, with prevalence reported between 7 and 30%; long standing diabetes results in a chronic low grade inflammatory state, and therefore can lead to exaggerated immune response as seen in COVID-19 infection [18]. Furthermore, diabetics have a higher usage of antibiotics due to frequent infections, hence their increased predisposition to CDI is due to gut dysbiosis [17,20]. Overall, our study also recognized diabetes to be the common factor amongst COVID-19 and CDI, and likely triggers increased morbidity and mortality when combined.

Chronic liver disease is known to be an independent risk factor for CDI as well as COVID-19 infection due to frequent hospitalizations and reduced immunity [21,22]. Immunologically, cirrhosis is characterized by reduction in the components of the complement system, compromised macrophage activation, impaired lymphocyte and neutrophil function; collectively known as cirrhosis-associated immune dysfunction (CAID) [22]. Moreover, CLD is also associated with changes in the gut microbiome and bile acid synthesis, both of which have been shown to increase CDI [23]. Keeping the above mechanisms in mind, multiple studies have shown that CDI and COVID-19 are individually associated with higher morbidity and mortality in patients with CLD [21,24,25,26]. Therefore, it was no surprise that that our study collectively found CDI and COVID-19 to have a positive association with CLD.

As discussed above, a compromised immune system plays a key role in predisposing patients to developing CDI and COVID-19, and similarly, our data analysis showed cancer to be more prevalent in the CDI and COVID-19 cohort (7.56% vs. 3.48%, *p* < 0.001). Unsurprisingly, multiple studies surrounding various malignancies individually demonstrated that CDI and COVID-19 can lead to overall poor outcomes [27,28,29]. Though these studies did not analyze CDI and COVID-19 in combination amongst cancer patients, it can be safely deduced that the combination of the two will yield even worse outcomes. Therefore, it is vital to maintain strict infectious disease prevention protocols of the highest standard in cancer wards.

The most common extrapulmonary complication in hospitalized COVID-19 patients appears to be AKI, reported in the literature to be at 25–30% [30,31], which is close to what we are reporting (28.5%), and interestingly, the rate increased with concurrent CDI up to 47.7%. Similarly, a study conducted on 2600 COVID-positive patients revealed a substantial increase in AKI requiring HD at 8.5% [32], whereas in our analysis we found 2.4%; when combined with CDI, it rose up to 7.7%. The cause of AKI could be due to direct tropism exerted by the virus to the renal parenchyma, leading to multiple complications such as acute tubular necrosis, interstitial nephritis, microvascular clots, or focal segmental glomerulosclerosis; although these etiologies seem to be less common [30,31]. Primarily, AKI is a result of secondary complications such as hemodynamic instability secondary to sepsis, respiratory failure, cytokine storm, or higher comorbidities [30,32,33]. In comparison, studies showed that CKD and ESRD patients are at a higher risk of acquiring CDI due to unknown mechanisms [34,35]. Furthermore, AKI in CDI is more often related to dehydration rather than a direct toxin effect [35]. Overall, the high incidence of renal injury in patients with simultaneous CDI and COVID-19 viral infection appears to be multifactorial, and the prevention of progression primarily hinges on prompt diagnosis and treatment of the underlying nephrotoxic etiology.

COVID-19 is one of the leading causes of ARDS, leading to increased mechanical ventilation in patients [36]. Mechanical ventilator use is itself a significant risk factor for contracting CDI [10]. Furthermore, CDI in mechanically ventilated patients leads to poor outcomes, increased length of stay, and higher in-hospital costs [37]. Similarly, patients with COVID-19 are at an increased risk of contracting nosocomial infections such as bacteremia, UTI, pneumonia and CDI, which leads to increased incidence of sepsis [38]. Presence of CDI alone in patients with septic shock leads to increased hospital length of stay and readmissions [39], when seen with COVID-19 infection it further increases morbidity and mortality. Our analysis showed increased prevalence of mechanical ventilation, intestinal perforation, and peritonitis in the CDI cohort, all of these are known to be direct causative complications for sepsis as seen in our analysis. Furthermore, a study looked at factors associated with complications of CDI such as ileus, perforation, colectomy and ICU admissions; found that older age, abnormal lab values and abnormal vital signs were the main factors associated with such complications [40], all these further validate our findings by showing positive associations amongst each other.

Our study showed a lower percentage of obesity in the CDI and COVID-19 cohort. There is no obvious explanation for it, however, in the literature, obesity was revealed to be associated with a lower risk of developing CDI [41,42]. Conversely, another study found obesity to be associated with acquiring CDI. Due to our study design, we cannot establish causality, and therefore the clinical significance of this finding at this point is unknown.

Multiple studies have provided evidence indicating that African Americans may face a greater risk of experiencing morbidity and mortality because of CDI, in comparison to individuals from other racial or ethnic groups. For instance, Argamany and colleagues found that while the incidence of CDI was higher in white individuals than in African Americans, the latter group experienced higher rates of mortality, longer hospital stays, and more severe cases of CDI compared to Caucasians. Moreover, their study identified African American race as a predictor of both mortality and severe CDI [43]. These results are very consistent with our analysis. There are several factors that may contribute to this increased risk, including differences in healthcare access, comorbidities, and microbiome composition. African Americans are more likely to experience healthcare disparities, including lower access to healthcare and lower quality of care [44]. African Americans are also more likely to have comorbidities, such as diabetes, hypertension, and chronic kidney disease, which may therefore increase the risk of severe CDI and mortality. These conditions can weaken the immune system and make it more difficult for the body to fight off infections [45]. Studies have shown that the composition of the gut microbiome may differ between African Americans and other racial or ethnic groups [46]. Differences in the gut microbiome may impact how the body responds to CDI, and how effective the treatments are in preventing severe complications and mortality. Additionally, it is important to note that African Americans may be more likely to be exposed to risk factors for CDI, such as prolonged hospitalization, use of antibiotics, and the use of proton pump inhibitors [47]. Overall, while the reasons for the increased risk of mortality from *C. difficile* infection in African Americans are not fully understood, it is important for healthcare providers to be aware of these disparities, and to take steps to ensure that all patients receive timely and appropriate care for CDI.

Antibiotic stewardship is an important strategy for mitigating the impact of COVID-19 on the healthcare system. While antibiotics are not effective against viruses such as SARS-CoV-2, they are often prescribed to treat bacterial co-infections or to prevent secondary infections in patients with COVID-19. However, the inappropriate use of antibiotics can lead to increased resistance, CDI, allergic reactions, and increased healthcare costs. Studies have shown that during pandemics, there is often an increase in the use of antibiotics, which can exacerbate the problem of antibiotic resistance [48]. Additionally, evidence suggests that COVID-19 patients are at an increased risk of developing healthcare-associated infections, including infections caused by antibiotic-resistant bacteria [49]. Therefore, implementing antibiotic stewardship practices, such as judicious antibiotic use and monitoring of prescribing practices, are crucial to prevent the development and spread of antibiotic resistance, and to ensure optimal patient outcomes during the COVID-19 pandemic [50].

COVID-19 and CDI together pose a significant threat to the ever-growing burden on our healthcare system. Our analysis not only proved a significant increase in LOS, but also revealed a consequent increased cost of total hospitalizations. Furthermore, we also observed that most of the patients with CDI and COVID-19 required some form of placement post discharge such as SNF/LTAC/nursing home, which further led to increased healthcare costs and load. All these findings were unanimously reported in the literature [14,21,37]. Therefore, from a financial and logistics standpoint, it is imperative to curb this rising incidence of CDI and COVID-19.

Despite numerous strengths of this study, specifically the big sample size from the NIS database, there are a few limitations. First, it is an observational study, mainly deriving its sample size from the NIS database, meaning it cannot prove causality. Moreover, it does not study the effects of CDI in COVID-19 patients in the outpatient setting, and consequently, the results cannot be extrapolated in the more stable outpatient setting. There is also a concern for detection bias since not everyone testing positive for COVID-19 underwent *C. difficile* testing, which may lead the results to be skewed in a certain direction. The NIS data, which relies on ICD-10 codes, does not include detailed information on patients’ antibiotic use, laboratory results, or other clinical parameters. As a result, we were unable to classify patients with CDI as having complicated or non-complicated CDI, which could have provided more nuanced insights into the differences between these subgroups. Additionally, the lack of data on specific treatments, such as bezlotoxumab, anti-CDI therapy (vancomycin, metronidazole, fidaxomicin, and tigecycline), and surgical therapy, precluded us from analyzing the impact of these interventions on patient outcomes. Similarly, we were unable to assess the recurrence rates of CDI among the study population. The study data primarily included non-vaccinated individuals, given the FDA first approved COVID vaccinations under EUA on 11 December 2020. Nevertheless, the large study size increases our study reliability by reducing the Type II error.

Despite these limitations, our study provides valuable information on the characteristics and outcomes of COVID-19 patients with and without CDI. Future research should aim to overcome these limitations by using more comprehensive data sources, such as electronic health records or clinical registries, which contain detailed clinical information on patients’ treatments, laboratory results, and disease progression. Such studies would enable a more in-depth analysis of the factors contributing to the development of CDI among COVID-19 patients, and could help identify potential risk factors, prognostic markers, and effective treatment strategies. Furthermore, future studies could also explore the impact of different anti-CDI therapies on patient outcomes, as well as the recurrence rates of CDI, to provide more actionable insights for clinical decision-making.

## 5. Conclusions

CDI was on the rise during the COVID-19 pandemic due to various reasons such as poor antibiotic stewardship and neglecting hand hygiene due to the overburdened healthcare system. We recommend adopting strict infection prevention protocols across all clinical settings as they are the biggest nidus for harboring *C. difficile* and contracting COVID-19 infection. Furthermore, CDI and COVID-19 in combination carry high morbidity and mortality, and therefore high-risk patients should be identified either via clinical or biochemical risk factors earlier in the disease course to prevent further decline. Additionally, we also recommend widespread vaccinations against the COVID-19 virus, especially amongst immunocompromised patients, along with encouraging antibiotic stewardship programs as well as supplementing pre-probiotics to help maintain a healthy gut microbiome, which may help in reducing CDI. Prompt *C. difficile* testing should be done in COVID-19 patients having diarrhea, as timely diagnosis and treatment initiation will lead to decreased complications such as ileus, perforation, sepsis, and AKI, amongst many others. Lastly, we also recommend aggressive in-patient physical and occupational therapy to improve functional status and early return to normal baseline, leading to improved length of stay and final disposition.

## Figures and Tables

**Table 1:** *Clostridioides difficile* and COVID-19 infection- unmatched patient-level characteristics.

Characteristics	COVID-19 Patients with *Clostridioides difficile* Infection N (%)	COVID-19 Patients without *Clostridioides difficile* Infection N (%)	*p*-Value
N = 1,659,040	N = 10,710 (0.64%)	N = 1,648,330 (99.35%)	
Sex (Female)	5610 (52.38%)	789,715 (47.91%)	0.001
Mean age years (SD)			
Male	67.6 (14.7)	63.3 (16.2)	
Female	70.4 (14.8)	63.01 (18.8)	
Age groups			<0.001
≥18–29	180 (1.68%)	81,758 (4.96%)	
30–49	955 (8.92%)	277,743 (16.85%)	
50–69	3615 (33.75%)	614,167 (37.26%)	
≥70	5960 (55.65%)	674,661 (40.93%)	
Race			<0.001
Caucasians	6369 (59.47%)	838,341 (50.86%)	
African American	2213 (20.67%)	313,842 (19.04%)	
Hispanics	1444 (13.48%)	354,720 (21.52%)	
Asian or Pacific Islander	226 (2.11%)	53,736 (3.26%)	
Native American	118 (1.1%)	16,978 (1.03%)	
Others	340 (3.17%)	70,713 (4.29%)	
Median household income			0.12
<49,999$	3388 (31.63%)	562,575 (34.13%)	
50,000–64,999$	3038 (28.37%)	448,180 (27.19%)	
65,000–85,999$	2511 (23.45%)	365,105 (22.15%)	
>86,000$	1773 (16.55%)	2,724,689 (16.53%)	
Insurance status			<0.001
Medicare	7733 (72.2%)	875,428 (53.11%)	
Medicaid	1280 (11.95%)	250,216 (15.18%)	
Private	1548 (14.45%)	457,081 (27.73%)	
Self-pay	149 (1.39%)	65,604 (3.98%)	
Hospital division			<0.001
New England	440 (4.11%)	62,472 (3.79%)	
Middle Atlantic	1550 (14.47%)	240,821 (14.61%)	
East-North Central	2035 (19%)	255,820 (15.52%)	
West-North Central	880 (8.22%)	110,932 (6.73%)	
South Atlantic	2145 (20.03%)	330,984 (20.08%)	
East-South Central	605 (5.65%)	110,768 (6.72%)	
West-South Central	1109 (10.36%)	236,371 (14.34%)	
Mountain	785 (7.33%)	113,899 (6.91%)	
Pacific	1160 (10.83%)	186,261 (11.3%)	
Hospital Bed size			<0.001
Small	2194 (20.49%)	401,368 (24.35%)	
Medium	2885 (26.94%)	478,016 (29%)	
Large	5630 (52.57%)	1,179,050 (46.64%)	
Hospital teaching status			0.24
Rural	960 (8.96%)	161,701 (9.81%)	
Urban non-teaching	1880 (17.55%)	307,578 (18.66%)	
Urban teaching	7870 (73.48%)	1,179,050 (71.53%)	
Comorbidities			
Coronary artery disease	2485 (23.2%)	295,546 (17.93%)	<0.001
Congestive heart failure	3380 (31.56%)	288,128 (17.48%)	<0.001
Hypertension	5220 (48.74%)	446,862 (27.11%)	<0.001
Diabetes mellitus	4990 (46.59%)	656,365 (39.82%)	<0.001
Chronic liver disease	960 (8.96%)	89,669 (5.44%)	<0.001
Cancer	810 (7.56%)	57,361 (3.48%)	<0.001
Chronic pulmonary disease	2550 (23.81%)	362,468 (21.99%)	0.04
Collagen vascular disorders	570 (5.32%)	47,968 (2.91%)	<0.001
Chronic kidney disease	4440 (41.46%)	338,732 (20.55%)	<0.001
Obesity	2070 (19.33%)	423,291 (25.68%)	<0.001
Smoking	2515 (23.48%)	422,302 (25.62%)	0.03

**Table 2:** In-hospital outcomes: *Clostridioides difficile* and COVID-19 infection.

Variable	COVID-19 Patients with *Clostridioides difficile* Infection N (%)	COVID-19 Patients without *Clostridioides difficile* Infection N (%)	Adjusted Odds Ratio ^1^	*p*-Value
In-hospital mortality (N = 222,490)	2464 (23.02%)	220,216 (13.36%)	1.3 (95% CI 1.15–1.46)	<0.001
Septic shock	2250 (21.01%)	119,009 (7.22%)	2.33 (95% CI 2.06–2.65)	<0.001
Acute kidney injury	5110 (47.71%)	469,939 (28.51%)	1.45 (95% CI 1.3–1.6)	<0.001
Acute kidney injury requiring HD	820 (7.66%)	39,724 (2.41%)	2.01 (95% CI 1.68–2.41)	<0.001
Mechanical ventilation	2994 (27.96%)	260,930 (15.83%)	1.42 (95% CI 1.27–1.58)	<0.001
Mean total hospitalization charge [USD(SD ^2^)]	196,012 (36,605)	91,162 (17,349)	USD 80,602	<0.001
Mean length of stay (days)	15.14	7.99	5.46 day higher	<0.001
Ileus	290 (2.71%)	12,527 (0.76%)		<0.001
Peritonitis	84 (0.79%)	2637 (0.16%)		<0.001
Fluid and electrolyte disorders	8004 (74.74%)	828,121 (50.24%)		<0.001
Intestinal perforation	45 (0.42%)	824 (0.05%)		<0.001
Ascites	264 (2.47%)	8406 (0.51%)		<0.001
Disposition				<0.001
Home/Routine	2315 (28.07%)	874,720 (61.25%)		
SNF ^3^/LTAC ^4^/Nursing home	4099 (49.72%)	314,613 (22.03%)		
Home health	1744 (21.15%)	219,258 (15.36%)		
AMA ^5^	88 (1.07%)	19,280 (1.35%)		

^1^ Adjusted for age, sex, race, income level, insurance status, discharge quarter, Elixhauser co-morbidities, hospital location, teaching status and bed size; ^2^ Standard deviation; ^3^ Skilled nursing facility; ^4^ Long-term acute care facility; ^5^ Against medical advice.

**Table 3:** Mortality breakdown in *Clostridioides difficile* and COVID-19 infection-unmatched sample.

Variable	COVID-19 Patients with *Clostridioides difficile* Infection N (%)	COVID-19 Patients without *Clostridioides difficile* Infection N (%)	*p*-Value
Total deceased (222,490)	2465	220,025	
Sex			<0.001
Male	1230 (49.9%)	128,846 (58.56%)	
Female	1235 (50.1%)	91,178 (41.44%)	
Age groups			
≥18–29	10 (0.41%)	1320 (0.6%)	0.56
30–49	145 (5.88%)	10,825 (4.92%)	0.32
50–69	770 (31.24%)	67,130 (30.51%)	0.73
≥70	1540 (62.47%)	140,727 (63.96%)	0.48
Race			
Caucasians	1425 (57.81%)	113,136 (51.42%)	0.006
African American	514 (20.89%)	370,008 (16.82%)	0.02
Hispanics	324 (13.18%)	43,257 (19.66%)	0.002
Asian or Pacific Islander	50 (2.03%)	7415 (3.37%)	0.09
Native American	35 (1.42%)	2684 (1.22%)	0.72
Others	65 (2.64%)	9725 (4.42%)	0.04

**Table 4:** In-hospital outcomes for 1:1 propensity-matched sample.

Variable	COVID-19 Patients with *Clostridioides difficile* Infection N (%)	COVID-19 Patients without *Clostridioides difficile* Infection N (%)	Adjusted Odds Ratio ^1^	*p*-Value
In-hospital mortality (N = 4035)	2325 (23.2%)	1710 (17.07%)	1.2 (95% CI 1.02–1.65)	0.02
Septic shock	2110 (21.06%)	915 (9.13%)	2.28 (95% CI 1.64–3.15)	<0.001
Acute kidney injury	4780 (47.7%)	3410 (34.03%)	1.59 (95% CI 1.25–2.01)	<0.001
Acute kidney injury requiring hemodialysis	755 (7.53%)	240 (2.4%)	2.18 (95% CI 1.22–3.9)	<0.001
Mechanical ventilation	2790 (27.84%)	1690 (16.87%)	1.43 (95% CI 1.08–1.9)	0.01
Mean total hospitalization charge [USD(SD ^2^)]	193,014 (26,490)	66,695 (8674)	USD 99,456	<0.001
Mean length of stay (days)	15.1	8.5	6.4 day higher	<0.001
Disposition				<0.001
Home/Routine	2148 (27.24%)	3413 (41.07%)		
SNF ^3^/LTAC ^4^/Nursing home	3929 (49.83%)	2922 (35.16%)		
Home health	1723 (21.86%)	1902 (22.89%)		
AMA ^5^	85 (1.08%)	73 (0.88%)		

^1^ Adjusted for age, sex, race, discharge quarter, income, insurance status, Elixhauser co-morbidities, hospital location, teaching status and bed size; ^2^ Standard deviation; ^3^ Skilled nursing facility; ^4^ Long-term acute care facility; ^5^ Against medical advice.

## Data Availability

The data used in this study are from the National (Nationwide) Inpatient Sample for the year 2020, obtained from the Healthcare Cost and Utilization Project. The NIS is a publicly available database, and researchers interested in accessing the data can obtain it directly from the HCUP website (https://www.hcup-us.ahrq.gov/ (accessed on 18 March 2023)).

## Supplementary Materials

The following supporting information can be downloaded at: https://www.mdpi.com/article/10.3390/idr15030028/s1, Table S1. ICD codes, Figure S1. Secondary analysis with propensity matching in COVID-19 patients with and without *Clostridium difficile* infection, Table S2. 1:1 Propensity matched variables in COVID-19 positive patients with and without *Clostridium difficile* infection: Patient-level characteristics (Age, sex, race, income and insurance status).
