# Supplementary material for: COVID-19 and Clostridioides difficile Coinfection Outcomes among Hospitalized Patients in the United States: An Insight from National Inpatient Database
Source: Infect Dis Rep. 2023 May 19;15(3):279–91. doi: 10.3390/idr15030028 (PMC10204388; doi:10.3390/idr15030028)
Supplement: Supplementary file 1 [file idr-15-00028-s001.zip › idr-2347175-supplementary.pdf]

Table S1. ICD codes.

Diagnosis	ICD-10 code
COVID 19	U071, U00, U49, U50, U85, J1282
Mechanical ventilation	5A1945Z,5A1955Z,5A1935Z,5A09357,5A09457,5A09557
vasopressor	3E030XZ,3E033XZ,3E040XZ,3E043XZ,3E050XZ,3E053XZ,3E060XZ,3E063XZ
Sudden cardiac arrest	I46, I97
Acute kidney injury and hemodialysis	N17, N990,5A1D70Z,5A1D90Z,5A1D80Z,5A1D00Z,5A1D60Z
Cardiogenic Shock	R570
Mechanical Circulatory Support	5A02110, 5A02210, 5A0211D, 02HA3RZ, 5A02116, 5A0221D, 5A1522F, 5A1522G, 5A1522H, 5A15A2F, 5A15A2G, 5A15A2H, 5A15223
Chronic kidney disease	N181, N182, N1830, N1831, N1832, N184,N185,N189
Pulmonary Circulation Disorder	Data Obtained from elixhauser comorbidity index
Chronic Pulmonary Disease	
Diabetes Uncomplicated	
Diabetes Complicated	
Hypothyroidism	
Peptic Ulcer Disease (excluding bleeding)	
Lymphoma	
Metastatic Cancer	
Solid Tumor Without Metastasis	
Rheumatoid Arthritis/Collagen Vascular	
Obesity	
Drug Abuse	
Hypertension	
Peripheral arterial disease	
Obstructive sleep apnea	
Liver Disease	
Alcohol	
Smoking	F17, F172, F1720, F17200, F17201, F17203, F17208, F17209, F1721, F17210, F17211, F17213, F17218, F17219, F1722, F17220, F17221, F17223, F17228, F17229, F1729, F17290, F17291, F17293, F17298, F17299, Z87891
History of PCI[1]	Z986, Z9861, Z9862
History of CABG[2]	Z951
Previous myocardial infarction	I252

Coronary artery disease	I2510, I25111, I25118, I25119, I252, I253, I254, I2541, I2542, I255, I256, I257, I2570, I25700, I25701, I25708, I25709, I2571, I25710, I25711, I25718, I25719, I2572, I25720, I25721, I25728, I25729, I2573, I25730, I25731, I25738, I25739, I2575, I25750, I25751, I25758, I25759, I2576, I25760, I25761, I25768, I25769,
Clostridium difficile infection	A04.7
Fluid & electrolyte disturbance	E87.0 - E87.8
Acute kidney injury	N17.9, N19
Septic shock	R65.21
Ileus	K56.0, K56.7
Ascites	R18.8
Hypoalbuminemia	E88.09
Peritonitis	K67, K65.0 - K65.2
Perforation of intestine	K63.1
Toxic megacolon	K52.1

[1] Percutaneous coronary intervention; [2] Coronary artery bypass graft

Figure S1. Secondary analysis with propensity matching in COVID-19 patients with and without Clostridium Difficile infection.

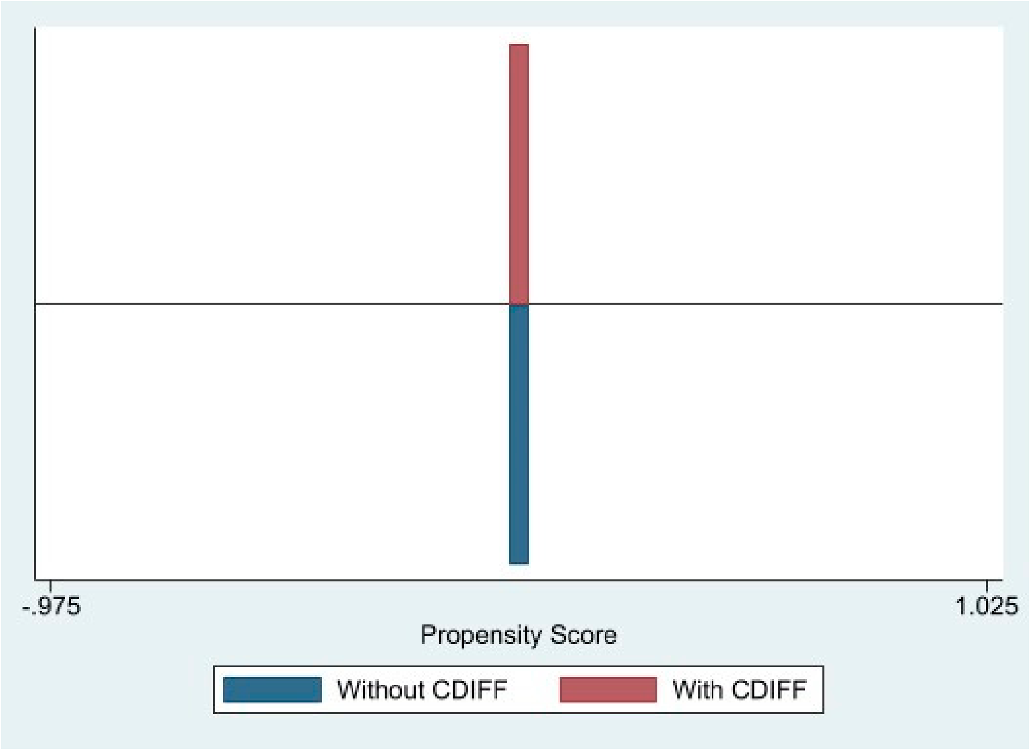

Baseline demographics (Age, race, sex, income status, and insurance status) were matched using a 1:1 nearest neighbor propensity score with 0.05 caliper width n matched cohort, and a secondary multivariate regression model was built

Table S2. 1:1 Propensity matched variables in COVID-19 positive patients with and without Clostridium Difficile infection: Patient-level characteristics (Age, sex, race, income and insurance status).

CHARACTERISTICS	COVID-19 patients with Clostridium Difficile infection	COVID-19 patients without Clostridium Difficile infection	P value
N=8170	N=10,020	N=10,020	
Sex (Female)	53.19%	53.14%	0.97
Mean age years (SD)			0.99
Male	68.02(11.06)	68.04(11.06)	
Female	70.7(11.11)	70.7(11.14)	
AGE GROUPS			0.99
≥18–29	1.55%	1.6%	
30–49	8.43%	8.43%	
50–69	33.53%	33.38%	
≥70	56.49%	56.59%	
RACE			0.99
Caucasians	59.93%	60.08%	
African American	20.66%	20.71%	
Hispanics	13.32%	13.32%	
Asian or Pacific Islander	2.05%	2%	
Native American	1%	0.85%	
Others	3.04%	3.04%	
MEDIAN HOUSEHOLD INCOME			0.99
<49,999$	31.29%	31.44%	
50,000–64,999$	28.34%	28.29%	
65,000–85,999$	23.9%	23.9%	
>86,000$	16.47%	16.37%	
INSURANCE STATUS			1
Medicare	72.51%	72.55%	
Medicaid	11.73%	11.68%	
Private	14.42%	14.42%	
Self-pay	1.35%	1.35%	
